# Nucleus‐specific RNAi nanoplatform for targeted regulation of nuclear lncRNA function and effective cancer therapy

**DOI:** 10.1002/EXP.20220013

**Published:** 2022-07-26

**Authors:** Zixian Huang, Shaomin Liu, Nan Lu, Lei Xu, Qian Shen, Zhuoshan Huang, Zhiquan Huang, Phei Er Saw, Xiaoding Xu

**Affiliations:** ^1^ Guangdong Provincial Key Laboratory of Malignant Tumor Epigenetics and Gene Regulation Guangdong–Hong Kong Joint Laboratory for RNA Medicine Medical Research Center Sun Yat‐Sen Memorial Hospital Sun Yat‐Sen University Guangzhou P. R. China; ^2^ RNA Biomedical Institute Sun Yat‐sen Memorial Hospital Sun Yat‐sen University Guangzhou P. R. China; ^3^ School of Medicine Sun Yat‐sen University Shenzhen P. R. China; ^4^ The Second Affiliated Hospital, Department of Clinical Pharmacology, Hengyang Medical School University of South China Hengyang P. R. China

**Keywords:** cancer therapy, LncRNA, nanoparticles, nucleus‐targeting, RNAi technology

## Abstract

In the context of cancer therapy, a recently identified therapeutic target is represented by the essential subtype of RNA transcripts ‐ the long noncoding RNAs (lncRNA). While this is the case, it is especially difficult to successfully regulate the expression of this subtype in vivo, particularly due to the protection granted by the nuclear envelope of nuclear lncRNAs. This study documents the development of a nucleus‐specific RNA interference (RNAi) nanoparticle (NP) platform for the targeted regulation of the nuclear lncRNA function, in order to effectuate successful cancer therapy. An NTPA (nucleus‐targeting peptide amphiphile) and an endosomal pH‐responsive polymer make up the novel RNAi nanoplatform in development, which is capable of complexing siRNA. The nanoplatform is capable of accumulating greatly in the tumor tissues and being internalized by tumor cells, following intravenous administration. The exposed complexes of the NTPA/siRNA may conveniently escape from the endosome with the pH‐triggered NP disassociation, following which it can target the nucleus by specifically interacting with the importin α/β heterodimer. In orthotopic and subcutaneous xenograft tumor models, this would result in a notable suppression of the expression of nuclear lncNEAT2 as well as greatly impede the growth of tumors in liver cancer.

## Graphical abstract

We herein developed a nucleus‐specific RNAi nanoplatform for targeted regulation of nuclear lncRNA function and effective cancer therapy. This new RNAi nanoplatform could respond to the endosomal pH to expose NTPA/siRNA complexes for the nucleus‐specific siRNA delivery, leading to a significant inhibition of nuclear lncNEAT2 expression and tumor growth of liver cancer.

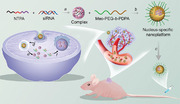

## 1 INTRODUCTION

With a length surpassing 200 nucleotides, long non‐coding RNAs (lncRNAs) have been identified as a novel subtype of RNAs that were previously dismissed as “transcriptional noise” or “junk DNA” due to their lack of ability to code proteins.^[^ ^1^ ^]^ However, with the recent advents and developments in the domain of molecular biotechnology, it has been proved that lncRNAs are also capable of effectuating their corresponding biological function by affecting various cellular behaviors by interacting with proteins, mRNA, and DNA.^[^ ^2^, ^3^, ^4^ ^]^ While a vast majority of the human genome is transcribed into several lncRNAs (>50%) that are primarily situated in the cytoplasm and nucleus; a negligible portion of it (<3%) is transcribed into protein‐coding mRNAs; the lncRNAs play a significant role in the progression of various diseases, cancer being one among these.^[^ ^5^ ^]^ Thus, while having been identified as a novel therapeutic target for the effective treatment of cancer, the challenge in the regulation of in vivo lncRNA has been an important concern that hampers their adoption in practical approaches.^[^ ^2^ ^]^

With the function of eliminating target genes, particularly the “undruggable” components of the human genome, technology that employs RNA interference (RNAi) has brought about a pivotal shift in the treatment of various diseases.^[^ ^6^ ^]^ Small interfering RNA (siRNA) among other similar RNAi effector molecules are capable of being incorporated within RNA‐induced silencing complex (RISC), a protein complex wherein Argonaute 2 (Ago2) may cleave the double strand of siRNA, while the target genes in the activated protein complex can be bound by its antisense strand, thereby starting to destroy the target genes—this is the fundamental mechanism that the technology operates upon.^[^ ^7^ ^]^ Due to the ability of the activated RISC to repeatedly degrade the target genes, this form of technology has been understood to be remarkable, potent, and characteristic of a prolonged preventive effect from several days (in dividing cells) to several weeks (in non‐dividing cells).^[^ ^8^ ^]^ Thus, for the regulated expression of long non‐coding RNAs as well as the overall progression of cancer, RNAi technology has been utilized across several practices associated with cancer therapy. Nevertheless, due to this intervention being generally compatible with long non‐coding RNAs situated within the cytoplasm; it is challenging to efficiently control the nuclear lncRNAs, even with the RISC located in the nucleus.^[^ ^9^ ^]^ Within the past decade, the employment of nanoparticles (NPs) for interventions with RNA, particularly in the case of stimuli‐responsive NPs capable of responding to endogenous factors in tumor cells/tissues to facilitate the delivery of siRNA,^[^ ^10^, ^11^ ^]^ there has been a noted efficiency in the successful silencing of gene targets in vivo. Yet, as a result of the protection granted by the nucleus envelope, there remain difficulties in silencing nuclear lncRNA expression.

**SCHEME 1:**
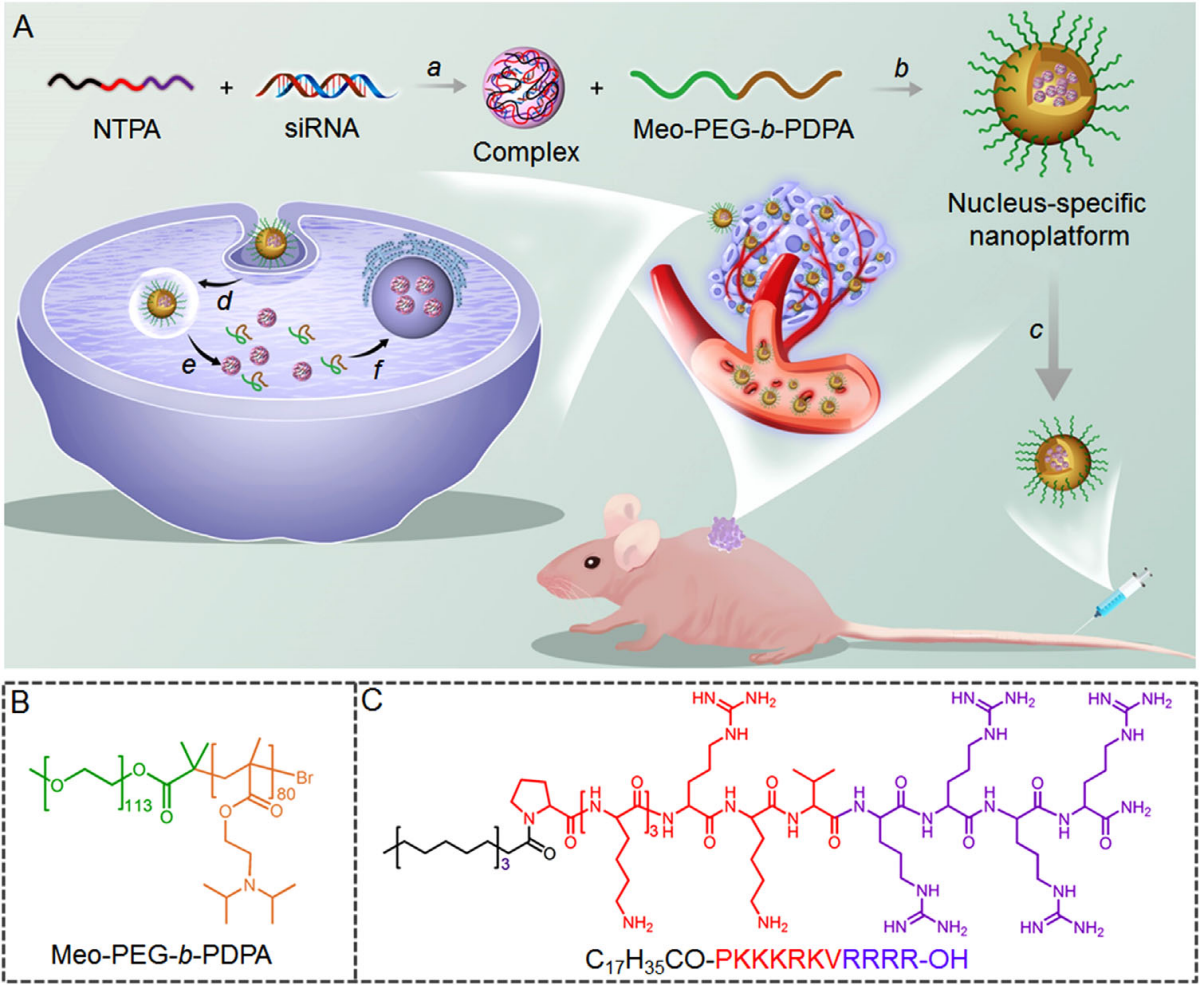
(A) The schematic illustration of nucleus‐specific RNAi nanoplatform for targeted regulation of nuclear lncRNA function and effective cancer therapy. In the newly developed nanoplatform, it was observed that NTPA, through electrostatic interaction, was capable of binding siRNA to form NTPA/siRNA complexes, (a) which are subsequently encapsulated within the hydrophobic cores of NPs composed of Meo‐PEG‐*b*‐PDPA, an endosomal pH‐responsive polymer. (b) During the systemic administering of the siRNA‐loaded NPs (c), they may accumulate in the tumor tissue after extravasating from leaky tumor vasculature. Following the process of internalization by tumor cells (d), a rapid NP disassociation and efficient endosomal escape of exposed NTPA/siRNA complexes is induced by the endosomal pH‐triggered protonation of hydrophobic PDPA chains (e), thereby, through the specific interaction with the importin α/β heterodimer, targeting the nucleus and achieving efficient nuclear siRNA transport. (f) This results in the effective silencing of the expression of nuclear lncRNA, also significantly inhibiting the growth of the tumor (B, C). The chemical structure of the endosomal pH‐responsive polymer Meo‐PEG‐*b*‐PDPA is shown by (B) and nucleus‐targeting peptide NTPA is shown in (C)

To rectify and overcome such a challenge, efforts have been made by the authors of this study toward the development of a nucleus‐specific RNAi nanoplatform for the targeted regulation of nuclear lncRNA function, which would lead to the effective treatment of cancer. The nanoplatform in question is composed of an endosomal pH‐responsive polymer, methoxyl‐poly(ethylene glycol)‐*b*‐poly(2‐(diisopropylamino)‐ethylmethacrylate) (Meo‐PEG‐*b*‐PDPA) as well as a nucleus‐targeting peptide amphiphile (NTPA) which, through electrostatic interaction, is capable of complexing siRNA. The novel platform exhibits the following characteristics for nucleus specific siRNA delivery, following siRNA encapsulation and intravenous administration (Scheme 1): (i) The outer shell of PEG has a prolonging effect on blood circulation, which facilitates the accumulation of the tumor;^[^ ^12^, ^13^ ^]^ (ii) a rapid NP disassociation as well as an efficient endosomal escape of siRNA/NTPA complexes (through the “proton sponge” effect) take place due to the endosomal pH‐triggered protonation of hydrophobic PDPA chains;^[^ ^14^, ^15^ ^]^ in addition to (iii), the specific interaction with the importin α/β heterodimer leads to the endowment of the abovementioned complexes with the ability to target nuclei due to the nuclear localization signal (NLS) peptide sequence (PKKKRKV) of NTPA.^[^ ^16^, ^17^ ^]^ As a proof‐of‐concept, we selected lncRNA nuclear enrichment autosomal transcript 2 (lncNEAT2) and examined the anticancer efficacy as well as systemic lncNEAT2 siRNA (siNEAT2) delivery through the developed nucleus‐specific nanoplatform. LncNEAT2, being a classic nuclear long non‐coding RNA, appears upregulated in different cancer types, thereby carrying the potential to promote the development of cancer by controlling the expression of different target genes such as c‐Myc, β‐catenin, and EZH2 at the transcriptional and posttranscriptional levels.^[^ ^2^, ^3^, ^18^ ^]^ According to the findings from our in vivo study, the newly developed nucleus‐specific nanoplatform was capable of delivering siNEAT2 efficiently to the nucleus and silencing the expression of lncNEAT2 expression, thereby majorly preventing the growth of tumor in liver cancer across orthotopic and xenograft tumor models.

## 2 MATERIALS AND METHODS

### 2.1 Preparation and characterizations of NPs

A homogenous solution was prepared by dissolving Meo‐PEG‐*b*‐PDPA in DMF, with a 10 mg/ml concentration. Following this preparation, a mixture of NTPA (5 mg/ml in DMF) and 1 nmol siNEAT2 (0.1 nmol/μl aqueous solution) with varying weight ratios was mixed with a 200 μl solution of Meo‐PEG‐*b*‐PDPA. The mixture was then added to deionized water (5 ml) dropwise, after vigorous stirring at 1000 rpm. So as to centrifuge and filter out the free compounds and organic solvents, the suspension was transferred to an ultrafiltration device (EMD Millipore, MWCO 100 K). The validated pH responsiveness of the siNEAT2‐loaded NPs received after washing it with 3 × 5 ml PBS (phosphate‐buffered saline) solution was suspended in a PBS solution of 1 ml with a pH of 7.4. With a NTPA/siNEAT2 weight ratio of 5, 10, 20, and 30, the siNEAT2‐loaded NPs were prepared and denoted NPs5, NPs10, NPs20, and NPs30. Using the instructions outlined above, control NPs were prepared with NTPA being altered with G0‐C14, a cationic lipid‐like compound. Using dynamic light scattering (DLS, Malvern, USA), the size and zeta potential were determined according to our previous methods.^[^ ^19^ ^]^ Furthermore, a transmission electron microscope was used to visualize the morphology of the NPs (Tecnai G^2^ Spirit BioTWIN). The sample had been stained with 1% uranyl acetate, then dried under air before observing. The Cy5‐siNEAT2‐loaded NPs were prepared in adherence to the method previously mentioned, and utilized to determine the EE% of siRNA. An NP suspension with a volume of 5 μl was extracted and mixed with 20‐fold DMSO. The standard was prepared by mixing 5 μl of naked Cy5‐siNEAT2 solution (1 nmol/ml in pH 7.4 PBS solution) with 20‐fold DMSO. By employing a Synergy HT multi‐mode microplate reader (BioTek, USA), the fluorescence intensity of Cy5‐siNEAT2 was evaluated, following which the EE% of siRNA was calculated as EE% = (FI_NPs_ / FI_Standard_) × 100.

### 2.2 Digestion assay

As per the method previously mentioned, the NPs that load fluorescein and Dabcyl‐labeled siNEAT2 were prepared and suspended in a PBS solution of 1 ml with a pH of 7.4. The sample was incubated at 37°C after 20 U of RNase was added. A microplate reader (480 nm) with an emission data range of 480–650 nm was used to evaluate the fluorescent emission spectra at predetermined time intervals.

### 2.3 In vitro siRNA release

NPs loaded with Cy5‐siNEAT2 were suspended in a PBS solution of 1 ml with a pH of 7.4 before being transferred to a PBS‐solution immersed (pH 7.4/6.0) Float‐a‐lyzer G2 dialysis device (MWCO 100 kDa, Spectrum) at 37°C. An amount of the NP suspension (5 μl) was extracted and mixed with 20‐fold DMSO at a predetermined interval. A microplate reader was used to determine the fluorescence intensity of Cy5‐siNEAT2.

### 2.4 Cell culture

A humidified atmosphere with 5 percent of carbon dioxide was ensured for the incubation of liver cancer cells, SK‐Hep1 and HepG2, in DMEM with 10% FBS at 37°C. The cell lines were free of mycoplasma contamination and were validated by short tandem repeat profiling analysis.

### 2.5 Confocal laser scanning microscopy (CLSM)

Glass bottomed dishes (#D35‐20‐0‐TOP, Cellvis) were used for seeding 10,000 SK‐Hep1 cells, which were later incubated for 24 h in DMEM (2 ml) with 10% FBS. A fresh medium of 2 ml replaced the previous medium, after which Cy5‐siNEAT2‐loaded NPs/Control NPs were added to the solution. The cells were placed in incubation at different times. The endosome and the nuclei were stained by Lysotracker green (#L7526, Thermofisher) and Hoechst (#33342, Thermofisher), respectively after the medium was removed and the items were washed thrice with PBS solution (pH 7.4). Finally, the intracellular distribution of Cy5‐siNEAT2 was observed using a ZEISS 800 CLSM.

### 2.6 Detection of Cy5‐siNEAT2 in the nuclei

Six‐well plates were used to seed 50,000 SK‐Hep1 cells, which were incubated for 24 h in DMEM (2 ml) with 10% FBS. A fresh medium of 2 ml replaced the previous medium, after which Cy5‐siNEAT2‐loaded NPs/Control NPs were added to the solution. The cells were incubated for a specific duration of 8 h. The cells were digested by trypsin, and a PARIS RNA isolation kit (#AM1921, Invitrogen) was used to collect the nuclei after the medium was removed and the items were washed with PBS solution (pH 7.4). The top solution was collected after repeated freezing (in liquid nitrogen) and subsequent thawing (at room temperature). Following this, a microplate reader was used to measure the fluorescence intensity of Cy5‐siNEAT2 in the nuclei.

### 2.7 Nuclear lncRNA silencing

Six‐well plates were used to seed 50,000 SK‐Hep1/HepG2 cells, which were incubated for 24 h in DMEM (2 ml) with 10% FBS. Following the placement of the medium with a fresh medium, the siNEAT2‐loaded NPs, Control NPs/lipofectamine 3000 (Lipo3k)/siNEAT2 complexes were added. The cells were washed with PBS solution (pH 7.4) after 24 h of incubation, after which they were incubated once again for 48 h in a fresh medium. Eventually, after trypsinizing the cells, Trizol was used to extract the total RNA from the cells. qRT‐PCR was used to evaluate the expression of lncNEAT2. Lysis buffer, which was supplemented with phenylmethanesulfonyl fluoride and protease inhibitor cocktail, was used to extract the total protein. Western blot was used to examine the expression of the c‐Myc protein. Cells were treated with the siPVT/siNEAT1‐loaded NPs to silence lncPVT1 and lncNEAT1. For qRT‐PCR analysis, the total RNA was extracted as well.

### 2.8 Apoptosis analysis

Six‐well plates were used to seed 50,000 SK‐Hep1 cells (per well), which were incubated for 24 h in DMEM (2 ml) with 10% FBS. After replacing the medium with a fresh one, NPs20, Control NPs/Lipo3k/siNEAT2 complexes were added at a siRNA dose of 30 nM. Following an incubation period of 24 h, PBS solution (pH 7.4) was used to wash the cells. Then, the cells were incubated further for 48 more hours in a fresh medium. After the cells were digested by trypsin, they were collected for the detection of annexin V and Propidium Iodide (PI) (Annexin V‐FITC/PI Kit, 40302ES20, Yeasen). A DXP11 Flow Cytometry Analyzer was used to perform the apoptosis analysis.

### 2.9 In vitro cell proliferation

Six‐well plates were used to seed 20,000 Sk‐Hep1 cells (per wall), which were incubated for 24 h in DMEM (2 ml) with 10% FBS. This step was followed by treatment of the cells with siNEAT2‐loaded NPs, Control NPs, or Lipo3k/siNEAT2 complexes at a siRNA dose of 30 nM for 24 h. A fresh medium replaced the previous medium after the cells were washed with PBS solution with a pH of 7.4. The cells were then incubated further. AlamarBlue assay was used to measure the cytotoxicity at predetermined intervals, followed by the removal of the measurement agent. Then, the cells were incubated for a longer time to observe the formation of the colony.

### 2.10 Animals

For the purpose of the study, nude mice and healthy female BALB/c normal mice aged around 4 or 5 weeks were purchased from the Sun Yat‐Sen University Experimental Animal Center. The protocol approved by the Institutional Animal Care and Use Committee at Sun Yat‐Sen University was adhered to for in vivo studies documented herein (SYSU‐IACUC‐2021‐B0690).

### 2.11 Pharmacokinetics study

Healthy female BALB/c mice were randomly divided into three groups (*n* = 3) and intravenously injected with either (i) free Cy5‐siNEAT2, (ii) Control NPs, or (iii) Cy5‐siNEAT2‐loaded NPs at a siRNA dose of 1 nmol per mouse. Orbital vein blood (20 μl) was withdrawn at various predetermined intervals utilizing a tube that contained heparin. The bleeding was stopped by pressing the wound for several seconds. A microplate reader was used to determine the fluorescence intensity of Cy5‐siNEAT2 in the blood.

### 2.12 Subcutaneous and orthotopic xenograft tumor model

Subcutaneous injection with 200 mL of SK‐Hep1 cell suspension (DMEM:Matrigel in a 1:1 volume ratio) with a density of 1 × 10^7^ cells/ml in the back region of healthy female nude mice was used to construct an SK‐Hep1 subcutaneous tumor model. The mice were used in the following in vivo experiments only when the volume of the tumor reached 70–100 mm^3^. Healthy nude mice were anesthetized with 2% pentobarbital with an opening made in the abdominal cavity that would expose the liver—this supported the establishment of the orthotopic tumor model. This was followed by the injection of luciferase (Luc)‐expressing SK‐Hep1 cells (1 × 10^6^ cells suspended in 30 μl of PBS solution). After closing the abdominal cavity, D‐luciferin substrate was injected intraperitoneally at a dose of 150 mg/kg. An IVIS Lumina III (Perkin‐Elmer, USA) imaging system was used to view the growth of the tumors in the mice. Bioluminescence imaging was used to evaluate the mean radiance of tumor sites.

### 2.13 Biodistribution

Mice that carried SK‐Hep1 subcutaneous tumors were divided randomly into three groups (*n* = 3), with each given an intravenous injection of either (i) free Cy5‐siNEAT2, (ii) Control NPs, or (iii) Cy5‐siNEAT2‐loaded NPs at a siRNA dose of 1 nmol per mouse. Following 24 h after the time of injection, In vivo imaging system (SmartChemi^TM^ 910plus, SINSAGE, China) was used to image the mice, following which organs and tumors were harvested and imaged. Image‐J was used to quantify the fluorescence intensity of each tissue; this measurement was used to assess the accumulation of Cy5‐siNEAT2 in the organs and tumors imaged.

### 2.14 Inhibition of subcutaneous tumor growth

Mice that carried SK‐Hep1 subcutaneous tumors were divided randomly into five groups (*n* = 5), with each given an intravenous injection (once every 2 days) of either (i) PBS, (ii) naked siNEAT2 (1 nmol siRNA dose for each mouse), (iii) Control NPs (1 nmol siRNA dose for each mouse), (iv) NPs loaded scrambled siRNA (1 nmol siRNA dose for each mouse), or (v) siNEAT2‐loaded NPs (1 nmol siRNA dose for each mouse). Four consecutive injections were administered to the mice, following which the growth of the tumor was monitored once every 2 days using a caliper to evaluate perpendicular diameters. The tumor volume was calculated as follows:

$$V=\;W^{2}\times \;L/2$$

where *W* and *L* are the shortest and longest diameters, respectively.

### 2.15 Inhibition of orthotopic tumor growth

Mice that carried SK‐Hep1 orthotopic tumors were divided randomly into five groups (*n* = 5), with each group being given an intravenous injection (once every 2 days) of either (i) PBS, (ii) naked siNEAT2 (1 nmol siRNA dose for each mouse), (iii) Control NPs (1 nmol siRNA dose for each mouse), (iv) NPs loaded scrambled siRNA (1 nmol siRNA dose for each mouse), or (v) siNEAT2‐loaded NPs (1 nmol siRNA dose for each mouse). Four consecutive injections were administered to all the mice. A bioluminescence imaging system was used to measure the growth of the tumor during the first week and at the end of the second week. All tumors were collected and sectioned at the end of the experiment for histological analysis.

### 2.16 Statistical analysis

The in vitro data were presented as mean ± S.D. of three independent experiments. Using a two‐tailed Student's *t‐*test that assumed equal variance, the statistical significance of the data was determined. Specifically, a *p*‐value < 0.05 is considered statistically significant in this study.

## 3 RESULTS AND DISCUSSION

### 3.1 Preparation and characterizations of nucleus‐specific NP platform

**FIGURE 1:**
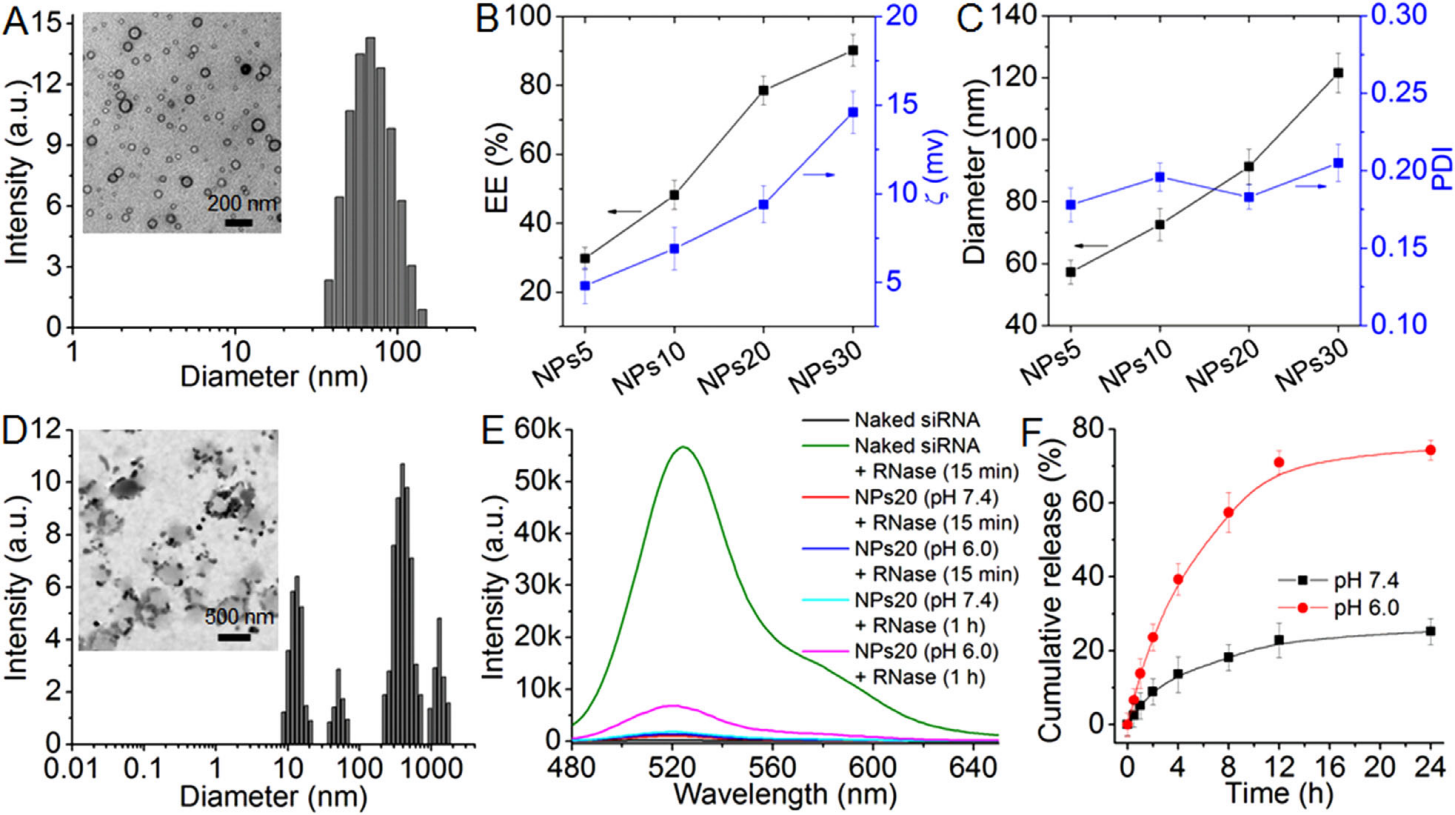
(A) Size distribution and morphology of NPs20 incubated in PBS solution at pH 7.4. (B,C) siRNA encapsulation efficiency (EE%), zeta potential (ζ), size, and polydispersity (PDI) of siNEAT2‐loaded NPs prepared at different weight ratios between NTPA and siNEAT2. (D) Size distribution and morphology of NPs20 incubated in PBS solution at 6.0. (E) Fluorescence emission spectra of NPs20 incubated in RNase‐containing PBS solution at different pHs. (F) Cumulative siNEAT2 release from the NPs20 incubated in PBS solution at different pHs

Through atom transfer polymerization, Meo‐PEG‐*b*‐PDPA (Scheme 1B) was synthesized as illustrated in Figures S1–S3. Using the classic acid‐base titration, the p*K_a_* of this polymer was found to be ∼6.34, which is proximally closer to the endosomal pH (6.0–6.5).^[^ ^20^, ^21^ ^]^ Given this data, the siNEAT2‐loaded NPs were prepared using the method of self‐assembly nanoprecipitation by mixing the DMF solution carrying Meo‐PEG‐*b*‐PDPA and NTPA (C_17_H_35_CONH‐PKKKRKVRRRR‐OH, Scheme 1C) with siNEAT2 aqueous solution. As observed in Figure 1A, it was possible for spherical NPs that are well defined with a mean size of ∼90 nm to be formed at an NTPA/siNEAT2 weight ratio of 20/1. The formation of NTPA/siNEAT2 complexes accompanied by the hydrophobic tails of NTPA molecules on the surfaces is induced by the electrostatic interaction in this self‐assembly system between the positively charged NTPA and the negatively charged siNEAT2. Upon mixing rapidly stirred water with the DMF mixture of Meo‐PEG‐*b*‐PDPA and NTPA/siNEAT2 complexes, the formation of NPs with the NTPA/siNEAT2 complexes embedded into the hydrophobic PDPA cores via hydrophobic interaction is induced by the spontaneous self‐assembly of amphiphilic polymer Meo‐PEG‐*b*‐PDPA.^[^ ^11^, ^19^ ^]^ By changing the NTPA/siNEAT2 weight ratio, the physiochemical characteristics of these NPs could be modified. According to the findings from the gel electrophoresis, the siNEAT2 was evidently encapsulated into the NPs (Figure S5). Furthermore, with an increase from 5 to 30 in terms of the weight ratio, the corresponding siNEAT2‐loaded NPs (denoted NPs5, NPs10, NPs20, and NPs30) demonstrated an increase in the siNEAT2 encapsulation efficiency from ∼29.8% to ∼90.2% (Figure 1B); with a rise in the average size, from ∼60 to ∼120 nm (Figure 1C). An explanation for such an occurrence may be associated with how stronger electrostatic interaction with siNEAT2 takes place with an increase in the weight ratio, leading to further entrapment of NTPA/siNEAT2 complexes within larger sized NPs. It must be noted that the gene‐silencing efficacy of NPs5 was not focused upon in subsequent experiments due to the relatively low siNEAT2 encapsulation efficiency (<30%).

Following preparation, the authors of this study evaluated the pH response of siNEAT2‐loaded NPs using NPs20 as an example. Using a PBS solution with a pH of 6.0, these NPs are incubated, after which a rapid NP dissociation takes place as a result of the protonated PDPA segment of polymer Meo‐PEG‐*b*‐PDPA (Figure S6),^[^ ^15^, ^20^ ^]^ thereby causing an alteration in the morphology, changing the structure from well‐defined spheres to large‐sized amorphous aggregates with tiny particles (Figure 1D). These may correspond respectively with the polymer and NTPA/siNEAT2 complexes. These findings are supported further by the DLS analysis shown in Figure 1D. In this analysis, after incubating NPs20 in PBS solution at pH 6.0 for a prolonged amount of time, it was possible to detect particles that ranged between several to thousand nanometers. In order to confirm the existence of NTPA/siNEAT2 complexes following the NP dissociation, modified siNEAT2 with fluorescein (FL) and its quencher (Dabcyl) were encapsulated into the NPs20; fluorescence of FL was evaluated in the presence of RNase. A significant rise in the fluorescence of FL is characterized by the separation of FL from its quencher, resulting in the rapid degradation of naked siNEAT2 by RNase within 15 min, as seen in Figure 1E. Upon incubating the NPs20 in the RNase‐containing PBS solution (pH 7.4) during the same time frame, the fluorescence of FL is found to be significantly weak, implying that the developed nanoplatform is capable of enhancing the stability of encapsulated siRNA. While NP dissociation was observed at pH 6.0 (Figure 1D), only a significantly weak fluorescence was noted, indicative of the presence of NTPA/siNEAT2 complexes post the pH‐triggered NP dissociation; also implying that the siNEAT2 could still be protected, by these exposed complexes, from being degraded by RNase. Nevertheless, there is an apparent increase in the FL fluorescence during incubation of the NPs20 in the solution with RNase (pH 6.0) for a prolonged period (e.g., 1 h). This is underpinned by a relatively faster siNEAT2 release, as opposed to the siNEAT2‐loaded NPs, which are incubated in a solution with a pH level of 7.4. While over 50% of the loaded siNEAT2 has been released (at a pH level of 6.0), lower than 20% of the loaded siNEAT2 was released from the NPs20 incubated for 8 h at a pH level of 7.4 (Figure 1F).

### 3.2 Evaluation of the nucleus‐targeting ability

**FIGURE 2:**
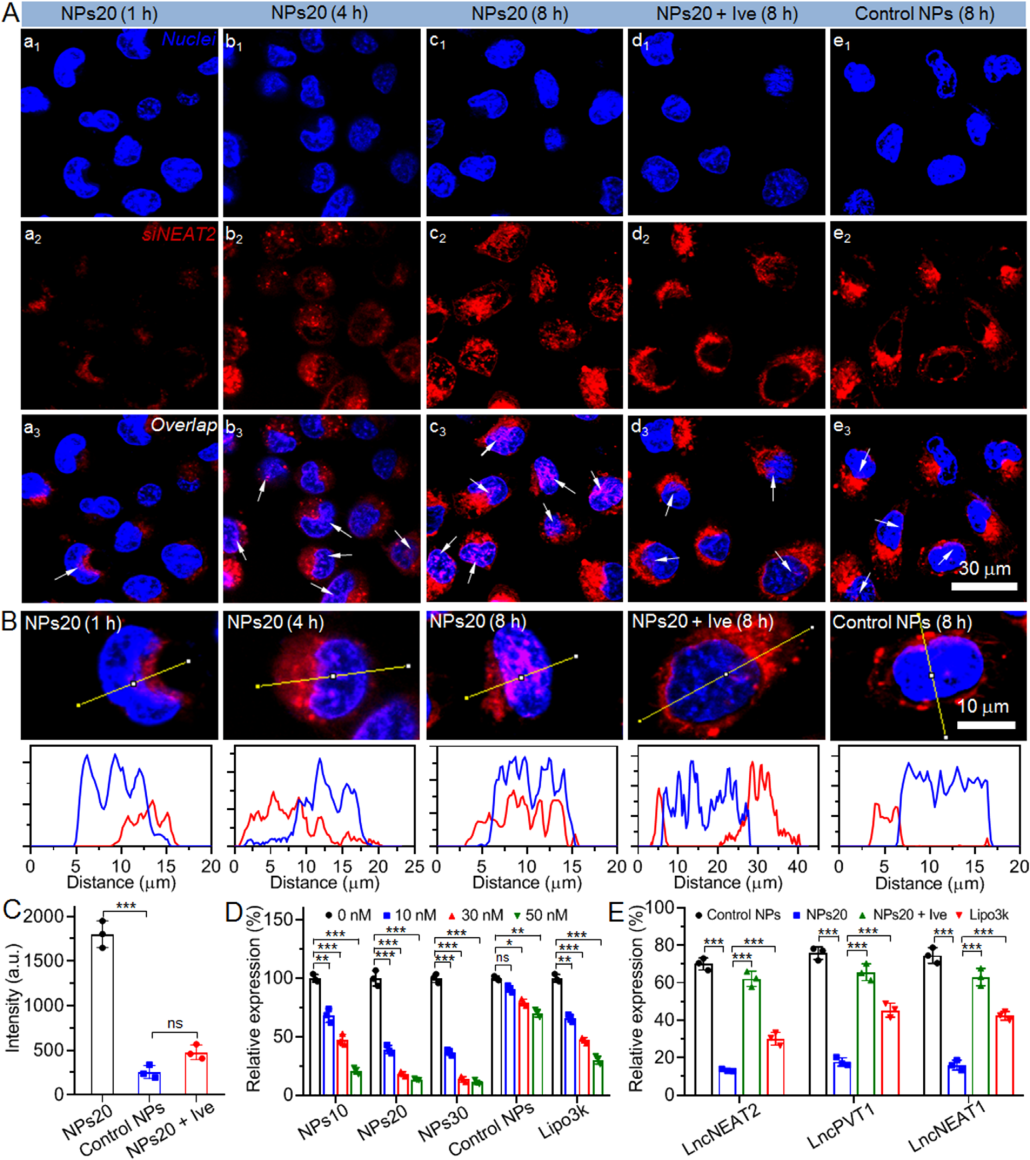
(A) CLSM images of SK‐Hep1 cells incubated with the NPs20 for 1, 4, 8 h; Control NPs for 8 h, and Ive for 2 h. This is followed by incubation of the NPs20 for 8 h at a siNEAT2 dose of 30 nM. Hoechst 33342 was used to stain the nuclei; in the nuclei, Cy5‐siNEAT2 has been indicated by white arrows. (B) Enlarged CLSM images and overlap between red fluorescence (Cy5‐siNEAT2) and blue fluorescence (nuclei) of SK‐Hep1 cells incubated with the formulas shown in (A). (C) Fluorescence intensity of Cy5‐siNEAT2 in the isolated nuclei of SK‐Hep1 cells incubated with the NPs20 for 8 h; Control NPs for 8 h, and Ive for 2 h, followed by the NPs20 for 8 h at a siNEAT2 dose of 30 nM, respectively. (D) A qRT‐PCR analysis of SK‐Hep1 cells treated with the siNEAT2‐loaded NPs, Control NPs, and Lipo3k/siNEAT2 complexes at different siNEAT2 doses, respectively, was used to determine the expression level of lncNEAT2. (E) A qRT‐PCR analysis of SK‐Hep1 cells treated with the NPs20, Control NPs, and Lipo3k/siRNA complexes at a siRNA dose of 30 nM, respectively, was used to determine the expression level of lncNEAT2, lncPVT1, and lncNEAT1. *ns*, no significance; *p < 0.05; **p < 0.01;***p < 0.001

The above evaluation was followed by a procedure carried out to assess the ability of siNEAT2‐loaded NPs to specifically deliver siRNA into the nucleus. For this purpose, fluorescent dye Cy5‐labeled siNEAT2 (Cy5‐siNEAT2) was encapsulated in the NPs20. This was followed by its incubation with SK‐Hep1. As illustrated by Figure 2A, which depicts the relevant CLSM images, it is clear that SK‐Hep1 was capable of internalizing the siNEAT2‐loaded NPs, which increases as the time is prolonged from an hour to 8 h (Figures 2Aa_2_ and 2Ac_2_, respectively). Significantly, it is important to note that accompanied by the endosomal pH‐triggered protonation of polymer Meo‐PEG‐*b*‐PDPA for the induction of endosomal swelling through the “proton sponge” effect,^[^ ^14^ ^]^ the encapsulated siNEAT2 enters the nuclei after conveniently escaping from the endosome (Figure S7). This has been demonstrated by the bright red fluorescence associated with Cy5‐siNEAT2 in the nuclei (Figure 2Ac_3_,B). In contrast, if replacing the nucleus‐targeting peptide NTPA with G0‐C14 (the previously prepared cationic lipid‐like compound) without nuclear specificity (Figure S8),^[^ ^19^ ^]^ the resulting siNEAT2‐loaded NPs (denoted as Control NPs) are majorly distributed in the cytoplasm. Only a small amount of encapsulated siNEAT2 is capable of entering the nuclei in this case (Figure 2Ae_3_,B). These results indicate that the exposed NTPA/siNEAT2 complexes after endosomal pH‐triggered NP dissociation could specifically enter the nucleus. More importantly, by labeling the NTPA with Alexa Fluor 488 succinimidyl ester, there is an apparent dissociation of green and red fluorescence (Figure S9), indicating that siNEAT2 could be released from the NPTA/siNEAT2 complexes in the nucleus. To further validate the nucleus‐targeting ability of NTPA, SK‐Hep1 cells were pre‐treated with 50 μM of ivermectin (Ive) for 2 h and then further incubated with the NPs20 for 8 h. NLS‐mediated nuclear transport has been known to primarily depend on its engagement with importin α/β heterodimer. For instance, the NLS peptide sequence is directly bound by importin α while importin β, via nuclear pores, mediates nuclear transport, of which Ive acts as a significant inhibitor.^[^ ^16^, ^22^ ^]^ While Ive treatment was not found to have impacted the cellular uptake of siNEAT2‐loaded NPs, it was noted that the treatment was notably capable of suppressing the nuclear transport of encapsulated siNEAT2 (Figure 2Ad_3_,B). These finds are confirmed by the detection of siNEAT2 fluorescence intensity within the isolated nuclei (Figure 2C), which was found to be ten times more robust in the nuclei of SK‐Hep1 cells treated with the NPs20, relative to cells treated using Control NPs/Ive. The abovementioned findings are demonstrative of the fact that the siRNA delivery can be enhanced through interactions between importin α/β heterodimer and the NLS peptide sequence of NTPA with the incorporation of NTPA into the siNEAT2‐loaded NPs.

### 3.3 In vitro silencing of nuclear lncRNA expression

Given the positive findings with regards to the nucleus targeting property that was outlined, it was then imperative to determine whether the expression of lncNEAT2 could be efficiently silenced by the siNEAT2‐loaded NPs; the target here is a typical nuclear long non‐coding RNA that is associated with poor prognosis of liver cancer (Figure S10).^[^ ^2^, ^3^, ^18^, ^23^ ^]^ Based on the siNEAT2 dose which determines the differential silencing efficacy, all siNEAT2‐loaded NPs were capable of down‐regulating the expression of lncNEAT2 (Figure 2D). In comparison to NPs10, both NPs20 and NPs30 demonstrated stronger efficiency in silencing target genes. Further, the expression of lncNEAT2 could be downregulated by approximately 80% at a siNEAT2 dose of 30 nM—a statistic much more significant and higher than found among cells treated with Control NPs or Lipo3k/siNEAT2 complexes. Such findings underline the implication that the silencing of nuclear lncRNA expression may be efficiently carried out by the property of the developed siNEAT2‐loaded NPs to target nuclei. NPs20 was selected for the experiments that followed as the developed RNAi nanoplatform was characteristic of low cytotoxicity (Figure S11), moderate zeta potential, small particle size (<100 nm), high silencing efficacy, and good stability (Figure S12). The authors of this study encapsulated the siRNA targeting lncRNA plasmacytoma variant translocation 1 (lncPVT1) and nuclear paraspeckle assembly transcript 1 (lncNEAT1) to support the findings that evidence the efficient gene silencing capability of NPs20, as they are the two recognized oncogenes emerging across various cancer types,^[^ ^2^, ^3^, ^18^ ^]^ the ability of these variables to silence lncPVT1 and lncNEAT1 expression was also assessed. One may see illustrated in Figure 2E that in comparison to Control NPs or Lipo3k/siRNA complexes, NPs20 were characteristic of higher gene silencing efficacy. Further, approximately 80% of lncPVT1/lncNEAT1 expression may be downregulated in SK‐Hep1 cells at a siRNA dose of 30 nM. The significant weakening of the gene silencing property of NPs20 as a result of the use of Ive for the impeding of importin α/β heterodimer‐dependent nuclear transport is highly indicative of the significance of the NLS peptide sequence of NTPA in the context of nucleus‐targeted siRNA delivery. The efficiency of the gene‐silencing property of NPs20 may also be identified in HepG2 cells, wherein it is possible to suppress over 75% expression of lncNEAT2, PVT1, or NEAT1 at a siRNA dose of 30 nM (Figure S13).

**FIGURE 3:**
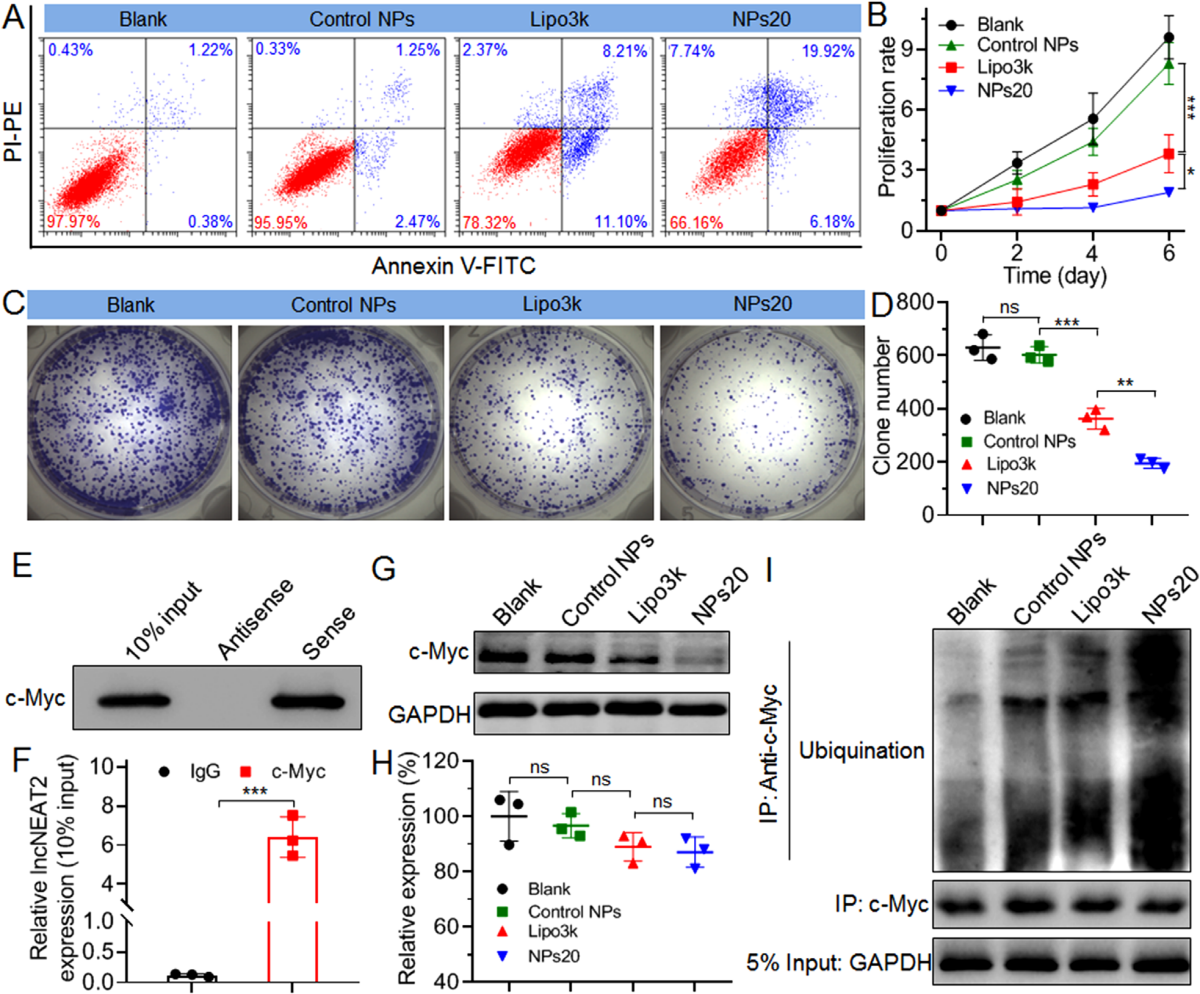
(A) Flow cytometry analysis of apoptosis of SK‐Hep1 cells treated with the NPs20, Control NPs, and Lipo3k/siNEAT2 complexes at a siNEAT2 dose of 30 nM, respectively. Proliferation profile (B), colony formation (C), and clone number (D) of SK‐Hep1 cells treated with the formulas shown in (A). (E) Western blot analysis of RNA‐protein binding complexes after RNA pulldown of lncNEAT2 in SK‐Hep1 cells. (F) RIP of c‐Myc in SK‐Hep1 cells. LncNEAT2 was retrieved by c‐Myc or IgG, followed by detection using qRT‐PCR. Protein (G) and mRNA level (H) of c‐Myc, respectively, was determined by western blot and qRT‐PCR analysis of SK‐Hep1 cells treated with the formulas shown in (A). (I) Western blot analysis of ubiquitination of c‐Myc protein in SK‐Hep1 cells was treated with the formulas shown in (A). The cells were pre‐treated with proteasome inhibitor MG132 (20 nM) for 4 h before collecting lysate. *ns*, no significance; *p < 0.05; **p < 0.01; ***p < 0.001

The above procedure was followed by an evaluation of the impact of lncNEAT2 silencing in association with the biological behaviors of SK‐Hep1 cells. For instance, it leads to highly evident apoptosis of SK‐Hep1 cells (Figure 3A). The rate of apoptotic cells (for cells treated with NPs20) reaches around 26% at a siRNA dose of 30 nM (Figure S14); a rate that is significantly much higher than among cells treated with Control NPs (∼3.7%) or Lipo3k/siNEAT2 complexes (∼19.3%). Considering such variation and improvements in the apoptosis, the multiplication of the SK‐Hep1 cells is greatly suppressed; further, a lower‐than‐twofold increase is identified within 6 days in terms of the number of cells treated with the NPs20 (Figure 3B). During the same period of time, however, for the cells treated with Control NPs or Lipo3k/siNEAT2 complexes, the cell count goes up 4‐ or even 8‐fold. A resembling phenomenon is observed in the outcome of the colony formation assay (Figure 3C,D), wherein NPs20, as compared to Control NPs or Lipo3k/siNEAT2 complexes, is demonstrative of a relatively higher ability in terms of inhibiting the colony formation of SK‐Hep1 cells.

Following this analysis, it was important to identify and examine the molecular mechanism that underpins the silencing ability of lncNEAT2. As per extant literature, lncNEAT2, rather than encoding protein, is capable of exerting its biological function, which manifests as the regulation of several oncogene expressions like c‐Myc, which occurred in over 70% of human cancers.^[^ ^23^, ^24^ ^]^ This study determined, using RNA pulldown assay and a subsequent western blot analysis if lncNEAT2 was capable of interacting with c‐Myc among SK‐Hep1 cells. In the lncNEAT2 pulldown complexes, c‐Myc protein is very prone to being greatly enriched, thus indicative of a highly specific interaction between the two (Figure 3E). As demonstrated in Figure 3F, this is confirmed by the use of antibodies through RNA immunoprecipitation (RIP) against c‐Myc, wherein the amount of lncNEAT2 binding with the protein is approximately fifty times higher than what binds with the control protein (IgG). Drawing from this data, it was important for the authors of this study to measure the impact of lncNEAT2 silencing on the expression of c‐Myc among SK‐Hep1 cells. According to the findings from the western blot analysis (Figure 3G), following the NPs20‐mediated silencing of lncNEAT2 expression at a siNEAT2 dose of 30 nM, the protein level of c‐Myc was found to have been suppressed by more than 85%. It is important to note, however, that no apparent change was noted in the mRNA level of c‐Myc (Figure 3H), which indicated that c‐Myc expression could be regulated by lncNEAT2 at a post‐transcriptional level.^[^ ^3^, ^18^ ^]^ In order to confirm such a hypothesis, the researchers examined the ubiquitination of c‐Myc protein, as it is possible to be degraded by the classic ubiquitin‐proteasome pathway.^[^ ^25^ ^]^ It has been illustrated in Figure 3I that after silencing of lncNEAT2, there is an improvement in the ubiquitination of c‐Myc protein, which is linked with the silencing efficacy of lncNEAT2. The outlined findings imply that through ubiquitination, lncNEAT2 is capable of binding c‐Myc protein, thereby furthering its stability. Moreover, the silencing of the lncNEAT2 expression may impede this interaction, which furthers the degradation of c‐Myc protein and results in enhanced apoptosis and suppressed multiplication of SK‐Hep1 cells.

### 3.4 In vivo gene silencing and anti‐tumor efficacy

**FIGURE 4:**
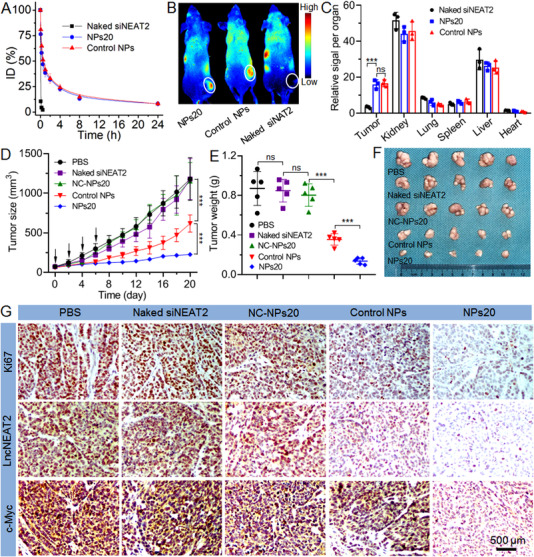
(A) The blood circulation profile of naked siNEAT2, NPs20, and Control NPs. (B) Overlaid fluorescence image of SK‐Hep1 subcutaneous tumor‐bearing mice 24 h after the injection of naked siNEAT2, NPs20, and Control NPs. Tumors have been indicated by ellipses. (C) Biodistribution of naked siNEAT2, NPs20, and Control NPs in the tumors and major organs of SK‐Hep1 xenograft tumor‐bearing mice were sacrificed 24 h following injection. Tumor size (D), tumor weight (E), and photographs of the collected tumors (F) of SK‐Hep1 subcutaneous tumor‐bearing mice treated with PBS, naked siNEAT2, Control NPs, NPs20, and NPs20 loading scrambled siRNA (NC‐NPs20). The intravenous injections are indicated by black arrows. (G) The expression of Ki67, lncNEAT2, and c‐Myc in the SK‐Hep1 tumor tissues after systemic treatment in each group. *ns*, no significance; ***p < 0.001

Given the desirable results obtained in vitro, the final procedure within this study was concerned with determining if the expression of lncNEAT2 can efficiently be silenced by delivering siRNA to tumor tissues via the newly developed RNAi nanoplatform for the inhibition of tumor growth. For healthy mice, an intravenous injection of NPs20 loading Cy5‐siNEAT2 was administered for the initial examination of pharmacokinetics (1 nmol siNEAT2 dose per mouse, *n* = 3). As one may observe in Figure 4A, the NPs20 and Control NPs exhibit prolonged blood circulation in comparison with naked siNEAT2 as a result of the protection granted by the PEG outer layer;^[^ ^12^ ^]^ additionally, 12 h after the injection, over 10% of injected NPs persist in the blood. Equipped with such a property, the NPs20 can greatly accumulate in the tumor tissues following intravenous injection into the SK‐Hep1 subcutaneous tumor‐bearing mice (1 nmol siNEAT2 dose per mouse, *n* = 3) (Figure 4B). Upon observing the fluorescence intensity of collected tumor tissues (Figure S15), the NPs20 demonstrated a tumor accumulation 6 times stronger than found in naked siNEAT2 (Figure 4C). Subsequently, for the evaluation of their anticancer effect, the NPs20 were intravenously injected into the SK‐Hep1 subcutaneous tumor‐bearing mice (1 nmol siNEAT2 dose per mouse, *n* = 5). Following the administering of four consecutive injections, tumor growth was shown to have been inhibited significantly, with a less than threefold increase in the size of the tumor (from ∼79 to ∼226 mm^3^) within 20 days (Figure 4D–F). In comparison, Control NPs exhibited a significant increase in the size of their tumors from ∼74 to ∼617 mm^3^, equivalent to an eightfold increase. The histological analysis illustrates the relatively more efficient anticancer effect of NPs20 (Figure 4G), in terms of proliferation inhibition, silencing of lncNEAT2 expression, and suppression of c‐Myc expression in tumor tissues.

**FIGURE 5:**
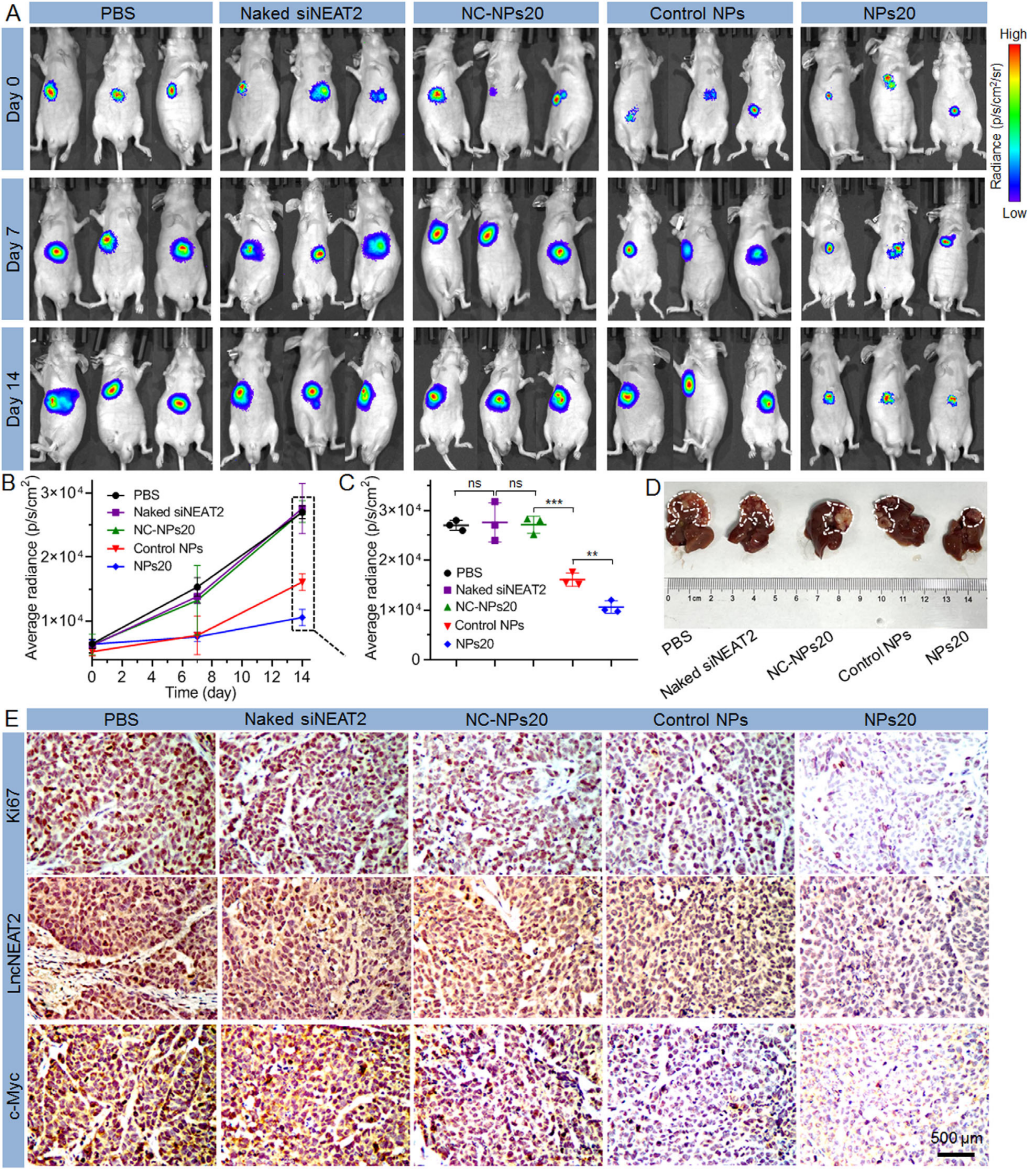
(A) Bioluminescence images of Luc‐SK‐Hep1 orthotopic tumor‐bearing mice treated with PBS, naked siNEAT2, Control NPs, NPs20, and NPs20 loading scrambled siRNA (NC‐NPs20). (B) Average radiance of tumor burden determined by bioluminescence imaging at day 0, 7, and 14. Average radiance of tumor burden (C) and photograph (D) of representative orthotopic tumors at the endpoint (day 14). Tumors are indicated by circled areas. (E) The expression of Ki67, lncNEAT2, and c‐Myc in the SK‐Hep1 orthotopic tumor tissues after systemic treatment in each group. *ns*, no significance; **p < 0.01; ***p < 0.001

By injecting Luc‐expressing SK‐Hep1 cells into the mouse liver followed by intravenous administering of NPs20 to the orthotopic tumor‐bearing mice every 2 days at a 1 nmol siRNA dose per mouse (*n* = 3), it was possible for us to establish an orthotopic liver cancer model to further evaluate the anticancer effect of NPs20. Using bioluminescence imaging, the average radiance of tumor tissues supported the monitoring of tumor growth (Figure 5A,B). As depicted in Figure 5C,D, the most efficient capability in reducing orthotopic tumor burden was demonstrated by the NPs20. All mice were euthanized at the end of the second week, after four rounds of treatment. This was followed by the collection of the orthotopic tumors for histological analysis (Figure 5E). The NPs20, once again, was demonstrative of the strongest ability to inhibit proliferation, silence lncNEAT2 expression, and suppress c‐Myc expression—results that paralleled the histological analysis of xenograft tumors (Figure 4G).

In subcutaneous and orthotopic tumor models, the administering of NPs20 was found to not affect mouse weight (Figure S16), suggestive of low in vivo toxicity of NPs20. Furthermore, NPs20 were intravenously injected into healthy mice (1 nmol siNEAT2 dose per mouse, *n* = 3) to sufficiently measure the potential in vivo side effects that correspond with it. The level of cytokines (Figure S17) and blood parameters (Figure S18) was found to be in the normal range following three daily injections. There was also no significant histological modification observed in the heart, liver, spleen, lung, and kidney tissues (Figure S19). Altogether, the results outlined above were indicative of low in vivo toxicity of NPs20.

## 4 CONCLUSION

A nucleus‐specific RNAi nanoplatform was developed herein for the targeted regulation of nuclear lncRNA function, which is bound to support and provide effective cancer therapy. Long blood circulation and high accumulation in tumor tissues are two primary properties associated with this newly developed RNAi nanoplatform. Following internalization by tumor cells, the protonation of pH‐responsive PDPA polymer in the endosome was capable of inducing rapid NP disassociation and efficient endosomal escape of exposed NTPA/siRNA complexes. This allowed for the subsequent targeting of the nucleus by leveraging the specific interaction between the NLS peptide sequence of NTPA and importin α/β heterodimer, which achieves nuclear siRNA transport. Altogether, this leads to the efficient suppression of nuclear lncNEAT2 expression and significant inhibition of tumor growth in liver cancer. The nucleus‐specific RNAi nanoplatform could be used as an effective tool to regulate the function of various nuclear lncRNAs for cancer therapy.

## CONFLICT OF INTEREST

The authors declare no conflict of interest.

## Supporting information



Additional supporting information can be found online in the Supporting Information section at the end of this article

## Data Availability

All data related to this work are present in the article and in the Supporting Information. Any other data associated with this work are available from the corresponding authors upon request.
