# Supplementary material for: Nucleus‐specific RNAi nanoplatform for targeted regulation of nuclear lncRNA function and effective cancer therapy
Source: Exploration (Beijing). 2022 Jul 26;2(5):20220013. doi: 10.1002/EXP.20220013 (PMC10191018; doi:10.1002/EXP.20220013)
Supplement: Supplementary file 1 — Additional supporting information can be found online in the Supporting Information section at the end of this article [file EXP2-2-20220013-s001.docx]

**Supporting Information**

*of*

Nucleus-specific RNAi nanoplatform for targeted regulation of nuclear lncRNA function and effective cancer therapy

Zixian Huang,^a,b^ Shaomin Liu,^a,c^ Nan Lu,^a,b^ Lei Xu,^a,b^ Qian Shen,^d^ Zhuoshan Huang,^a,b^ Zhiquan Huang,^a,b^ Phei Er Saw ^a,b,d^* and Xiaoding Xu^a,b,d^*

*^a^* Guangdong Provincial Key Laboratory of Malignant Tumor Epigenetics and Gene Regulation, Medical Research Center, Sun Yat-Sen Memorial Hospital, Sun Yat-Sen University, Guangzhou, China

*^b^* RNA Biomedical Institute, Sun Yat-Sen Memorial Hospital, Sun Yat-Sen University, Guangzhou 510120, P. R. China

*^c^* School of Medicine, Sun Yat-Sen University, Shenzhen 518107, PR China

*^d^* The Second Affiliated Hospital, Department of Clinical Pharmacology, Hengyang Medical School, University of South China, Hengyang, 421001, PR China

*Correspondence: xuxiaod5@mail.sysu.edu.cn; caipeie@mail.sysu.edu.cn

**1. Materials**

Methoxyl-polyethylene glycol (Meo-PEG_113_-OH) was purchased from JenKem Technology. 2-(Diisopropyl amino) ethyl methacrylate (DPA-MA), α-bromoisobutyryl bromide, tetrahydrofuran (THF), triethylamine (TEA), *N,N,N',N',N'*-pentamethyldiethylenetriamine (PMDETA), copper (I) bromide (CuBr), N,N'-dimethylformamide (DMF), tetraethylenepentamine (TEPA), isopropyl alcohol, and dichloromethane (DCM) and other chemicals of laboratory grade were acquired from Sigma-Aldrich. Nucleus-targeting peptide-amphiphile (NTPA, sequence: C17H35CONH-PKKKRKVRRRR-OH) was obtained from GL Biochem Ltd. Cationic lipid-like compound alkyl-modified polyamidoamine (PAMAM) dendrimer (G0-C14) was synthesized according our previous studies. Lipofectamine 3000 (Lipo3K) was purchased from Invitrogen. The siRNA targeting lncNEAT2, lncPVT1, and lncNEAT1 were acquired from IGE Bio. The siRNA sequences are as follows: siNEAT2, 5’-GAG CAA AGG AAG UGG CUU ATT-3’ (sense) and 5’-UAA GCC ACU UCC UUU GCU CTT-3’ (antisense); siPVT1, 5’-GCU GAG AGG GUU GAG AUC UCU GUUU-3’ (sense) and 5’-CGA CUC UCC CAA CUC UAG AGA CAA A-3’ (antisense); siNEAT1, 5’-GUG AGA AGU UGC UUA GAA ACU UUC C 3’ (sense) and 5’-CAC UCU UCA ACG AAU CUU UGA AAG G-3’ (antisense). Cy5-labled siNEAT2 was purchased from IGE Bio and Cy5 was labeled at the 5’-end of both strands. Fluorescein and its quencher (Dabcyl)-labeled siNEAT2 was purchased from Guangzhou RiboBio Co. Ltd. Fluorescein was labeled at the 5’-end of the sense strand and Dabcyl was labeled at 3’-end of the antisense strand. Cell culture media , penicillin-streptomycin, trypsin, and fetal bovine serum (FBS) were purchased from Invitrogen Corp.. All other reagents and solvents are of analytical grade and used without further purification.

**2. Primers and antibodies**

The primers for reverse transcription quantitative polymerase chain reaction (qRT-PCR) are as follows: lncNEAT2, 5’-GCT TGA GAA GAT GAG GGT GTT T-3’ (forward sequence), 5’- TCC AAA AGC CTT CTG CCT TA-3’ (reverse sequence); lncPVT1, 5’-TGA GAA CTG TCC TTA CGT GAC C-3’ (forward sequence), 5’-AGA GCA CCA AGA CTG GCT CT-3’ (reverse sequence); lncNEAT1, 5’-CTT CCT CCC TTT AAC TTA TCC ATT CAC-3’ (forward sequence), 5’-CTC TTC CTC CAC CAT TAC CAA CAA TAC-3’ (reverse sequence). The information of antibodies is as follows: c-Myc rabbit mAb (#ab32072) was purchased from Abcam. Anti-rabbit IgG horseradish peroxidase (HRP)-linked secondary mAb (#7074) was purchased from Cell Signaling Technology (CST). Click-iTTM TUNEL Colorimetric IHC Detection Kit (#C10625) was obtained from ThermoFisher.

**3. Synthesis of Meo-PEG-Br**

Meo-PEG_113_-OH (8 g, 1.6 mmol) and TEA (1.3 mL, 9.6 mmol) were dissolved in 250 mL of DCM. In an ice-salt bath, α-bromoisobutyryl bromide (l mL, 8 mmol) dissolved in 10 mL of DCM was added dropwise. After stirring for 24 h, the mixture was washed with 1 M NaOH (3 × 50 mL), 1 M HCl (3 × 50 mL), and deionized water (3 × 50 mL), respectively. After drying over anhydrous MgSO4, the solution was concentrated, and cold ether was added to precipitate the product. After re-precipitation thrice, the product was collected as white powder after drying under vacuum. The synthesis of Meo-PEG-Br is shown in Fig. S1. The ^1^HNMR spectrum of Meo-PEG-Br is shown in Fig. S2.

**4. Synthesis of methoxyl-polyethylene glycol-*b*-poly (2-(diisopropylamino) ethylmethacrylate) (Meo-PEG-*b*-PDPA)**

The Meo-PEG-*b*-PDPA polymer was synthesized by atom transfer radical polymerization (ATRP) according to previous studies [1-3]. DPA-MA (2.6 g, 12 mmol), Meo-PEG-Br (0.75 g, 0.15 mmol), and PMDETA (31.5 μL, 0.15 mmol) were added to a polymerization tube. DMF (3 mL) and 2-propanol (3 mL) were then added to dissolve the monomer and initiator. After three cycles of freeze-pump-thaw to remove oxygen, CuBr (21.6 mg, 0.15 mmol) was added under nitrogen atmosphere and the polymerization tube was sealed under vacuum. After polymerization at 40 ^o^C for 24 h, tetrahydrofuran (THF) was added to dilute the product, which was then passed through a neutral Al_2_O_3_ column to remove the catalyst. The resulting THF solution was concentrated and the residue was dialyzed against THF, followed by deionized water. The polymer was collected as a white powder after freeze-drying under vacuum. The synthesis of Meo-PEG-b-PDPA is shown in Fig. S1. The ^1^HNMR spectrum is shown in Fig. S3. The molecular weight was determined by gel permeation chromatography (GPC) using THF as eluent. *M_n, GPC_* = 2.37 × 10^4^ (PDI = 1.22); *M_n,NMR_* = 2.21 × 10^4^.

**5. ^1^H Nuclear magnetic resonance (^1^HNMR)**

The ^1^HNMR spectra of the polymers were recorded on a Mercury VX-300 spectrometer at 400 MHz (Varian, USA), using CDCl_3_ as a solvent and TMS as an internal standard.

**6. Gel permeation chromatography (GPC)**

Number- and weight-average molecular weights (*M_n_* and *M_w_*, respectively) of Meo-PEG-*b*-PDPA polymer were determined by gel permeation chromatographic system equipped with a Waters 2690D separations module and a Waters 2410 refractive index detector. THF was used as the eluent at a flow rate of 0.3 mL/min. Waters millennium module software was used to calculate molecular weight based on a universal calibration curve generated by polystyrene standard of narrow molecular weight distribution.

**7. Acid-base titration**

The Meo-PEG-*b*-PDPA polymer was dispersed in deionized water, and a concentrated HCl aqueous solution was added until the copolymer was completely dissolved (1 mg/mL). Subsequently, 1 M NaOH aqueous solution was added in 1-5 μL increments. After each addition, the solution was constantly stirred for 3 min, and the solution pH was measured using a pH meter. The *pK_a_* of Meo-PEG-*b*-PDPA polymer was determined as the pH at which 50% of polymer turns ionized.

**8. qRT-PCR**

Total RNA was extracted from the cultured cells using Trizol and 1 μg of RNA was then reverse transcribed into cDNAs using a Superscript First-Strand cDNA Synthesis Kit (18080-051, Invitrogen, USA). qRT-PCR analysis was performed using SYBR Premix Ex Taq II kit (DRR081A, TAKARA, Japan) on a LightCycler 480 System (Roche, Switzerland).

**9. RNA Pulldown Assay**

The 3’-end biotin-labeled lncNEAT2 was first transcribed using T7 High Yield Transcription Kit (AM1334, Ambion, USA) and then purified with MEGAclear Kit (AM1908, Ambion, USA) according to the manufacturer’s instructions. Subsequently, 5 pmol of purified RNA was withdrawn and heated to 95 °C for 5 min and then placed at room temperature to allow the formation of proper secondary structure. This folded RNA was then incubated with the lysates of the cultured cells treated with lysis buffer supplemented with anti-RNase and protease/phosphatase inhibitor cocktail. One hour later, Dynabeads Streptavidin magnetic beads (65801D, Invitrogen, USA) were added and the mixture was allowed to shake at room temperature for 5 min. Subsequently, the magnetic beads were isolated and washed with lysis buffer (3 × 5 min). The RNA-protein binding complexes on the magnetic beads were finally added to the SDS-PAGE gel and separated by gel electrophoresis according to the protocol described below.

**10. Western blot**

Equal amounts of proteins, as determined with a bicinchoninic acid (BCA) protein assay kit (Pierce/Thermo Scientific) according to the manufacturer’s instruction, were added to sodium dodecyl sulfate polyacrylamide gel electrophoresis (SDS-PAGE) gels and separated by gel electrophoresis. After transferring the protein from gel to polyvinylidene difluoride (PVDF) membrane, the blot was blocked with 3% bovine serum albumin (BSA) in PBS solution containing 0.1% Tween 20 (PBST) for 1 h. Subsequently, the primary antibody was added to incubate with the blot at 4 ^o^C overnight. After washing the blot with PBST thrice, anti-rabbit IgG HRP-linked secondary antibody was added to incubate with the blot at 4 ^o^C for 1 h. The expression of c-Myc protein was detected using an enhanced chemiluminescence detection system after washing the blot with PBST thrice.

**11. RNA immunoprecipitation (RIP)**

The RIP assay was performed using the Magna RIP RNA-Binding Protein Immunoprecipitation Kit (17-700, Millipore, USA) according to the manufacturer’s instructions. In brief, lysates of the cultured cells were incubated with magnetic beads with 5 μg of IP-grade antibody and incubated in IP buffer at 4 ^o^C overnight. Subsequently, the RNA was collected, purified, and finally quantified by qRT-PCR. Input control and normal anti-rabbit IgG control were also tested to ensure the accuracy of detected signals from the protein-bound RNA.

**12. *In situ* hybridization (ISH)**

LncNEAT2 expression in the paraffin-embedded tumor tissues was examined using an ISH Optimization Kit (Roche, Switzerland) according to the manufacturer’s instructions. In brief, the tumor slides were treated with pepsin for 10 min at room temperature and incubated with 500 nM of digoxigenin (DIG)-labeled probe targeting lncNEAT2 (RiboBio, China) at 55 °C for 4 h. After washing with phosphate buffered saline containing 0.1% Tween 20 (PBST) (3 × 5 min) and blocking with 10% FBS for 30 min, the slices were incubated with secondary anti-digoxigenin (anti-DIG) antibody at 4 ^o^C overnight. Subsequently, the slides were washed with PBST (3 × 5 min) and incubated with the anti-rabbit IgG HRP-linked antibody for 1 h. After adding diaminobenzidine and hematoxylin, the slides were finally viewed under an optical microscope.

**13. Immunohistochemistry (IHC)**

IHC staining was performed on formalin-fixed paraffin-embedded tumor sections. Briefly, tumor slides were first heated to 60 ^o^C for 1 h, desparaffinized with xylene (3 × 5 min), and washed with different concentrations of alcohol. After retrieval of antigen using DAKO target retrieval solution at 95-99 ^o^C for 40 min, followed by washing, the slides were blocked with peroxidase blocking buffer (DAKO Company) for 5 min. After washing DAKO buffer the slides were incubated with c-Myc rabbit antibody diluted in DAKO antibody solution for 1 h. The slides were then washed and incubated with peroxidase-labeled polymer for 30 min. After washing and staining with DAB+ substrate-chromogen solution and hematoxylin, the slides we remounted and viewed under a MVX10 MacroView Dissecting scope equipped with OlympusDP80 camera.

**14. Immune response**

Healthy BALB/c mice were randomly divided into five groups (n = 3) and given an intravenous injection of either (i) PBS, (ii) naked siNEAT2 (1 nmol siRNA dose per mouse),, (iii) Control NPs (1 nmol siRNA dose per mouse), (iv) NPs loaded scrambled siRNA (NC-NPs20, 1 nmol siRNA dose per mouse), or (v) NPs20 (1 nmol siRNA dose per mouse). After three daily injections, the blood was collected at 24 h post the final injection and serum isolated for measurements of representative cytokines (TNF-α, IL-6, IL-12, and IFN-γ) by enzyme-linked immunosorbent assay or ELISA (PBL Biomedical Laboratories and BD Biosciences) according to the manufacturer’s instructions.

**15. Blood and histological analysis**

Healthy BALB/c mice were randomly divided into five groups (n = 3) and given an intravenous injection of either (i) PBS, (ii) naked siNEAT2 (1 nmol siRNA dose per mouse),, (iii) Control NPs (1 nmol siRNA dose per mouse), (iv) NPs loaded scrambled siRNA (NC-NPs20, 1 nmol siRNA dose per mouse), or (v) NPs20 (1 nmol siRNA dose per mouse). After three daily injections, the blood was collected 24 h post the final injection and serum isolated for measurements of representative blood parameters (AST, ALT, albumin, ALKP, BUN, creatinine, and total protein). Simultaneously, the main organs were collected, fixed with 4% paraformaldehyde, embedded in paraffin, and finally sectioned for hematoxylin-eosin (H&E) analysis.

**References**

[1] Zhou, K.; Wang, Y.; Huang, X.; Luby-Phelps, K.; Sumer, B. D.; Gao, J. *Angew. Chem. Int. Ed.* **2011**, 50, 6109-6114.

[2] Xu, X.; Wu, J.; Liu, Y.; Yu, M.; Zhao, L.; Zhu, X.; Bhasin, S.; Li, Q.; Ha, E.; Shi, J.; Farokhzad, O. C. *Angew. Chem. Int. Ed.* **2016**, 55, 7091-7094.

[3] Xu, X.; Wu, J.; Liu, Y.; Saw, P. E.; Tao, W.; Yu, M.; Zope, H.; Si, M.; Victorious, A.; Rasmussen, J.; Ayyash, D.; Farokhzad, O. C.; Shi, J. *ACS Nano* **2017**, 11, 2618-2627.

[4] X. Xu, J. Wu, S. Liu, P.E. Saw, W. Tao, Y. Li, L. Krygsman, S. Yegnasubramanian, A.M. De Marzo, J. Shi, C.J. Bieberich, O.C. Farokhzad, Redox-responsive nanoparticle-mediated systemic RNAi for effective cancer therapy, Small 14(41) (2018) 1802565.

[5] X. Zhu, Y. Xu, L.M. Solis, W. Tao, L. Wang, C. Behrens, X. Xu, L. Zhao, D. Liu, J. Wu, N. Zhang, I.I. Wistuba, O.C. Farokhzad, B.R. Zetter, J. Shi, Long-circulating siRNA nanoparticles for validating Prohibitin1-targeted non-small cell lung cancer treatment, Proc. Natl. Acad. Sci. USA. 112(25) (2015) 7779-7784.





**Fig. S1.** Synthesis route of Meo-PEG-Br and Meo-PEG-*b*-PDPA polymer.





**Fig. S2.** ^1^HNMR spectrum of Meo-PEG-Br in CDCl_3_.





**Fig. S3.** ^1^HNMR spectrum of the Meo-PEG-*b*-PDPA polymer in CDCl_3_.





**Fig. S4.** Acid-base titration profile of Meo-PEG-*b*-PDPA polymer.


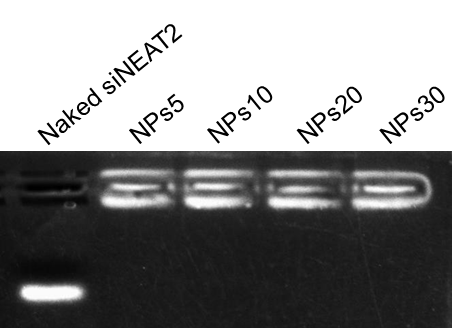


**Fig. S5.** Agarose gel electrophoresis retardation assay of naked siNEAT2 and siNEAT2-loaded NPs.





**Fig. S6.** NP number (count rate) of NPs20 in PBS buffer at pH 6.0 for different times.

**
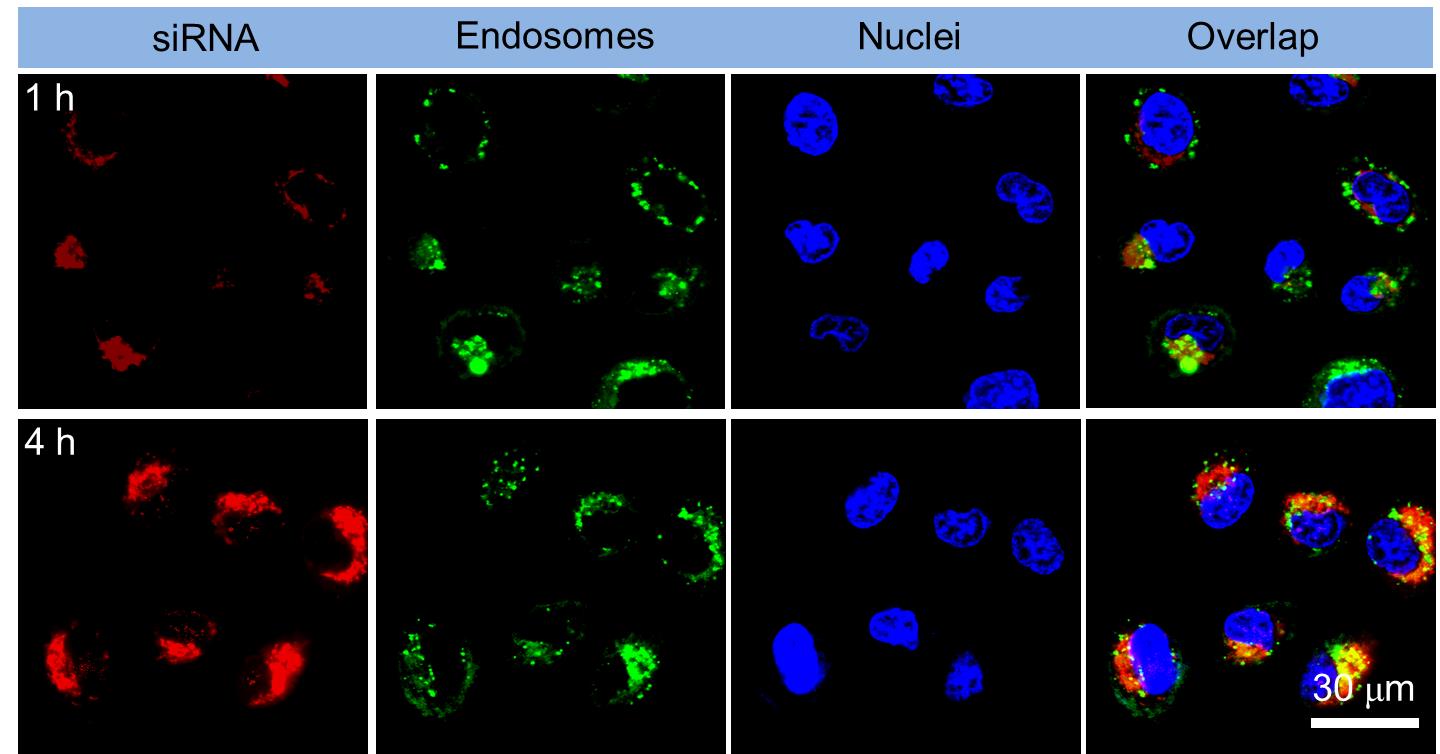
**

**Fig. S7.** CLSM images of SK-Hep1 cells incubated with NPs20 for 1 and 4 h, respectively.


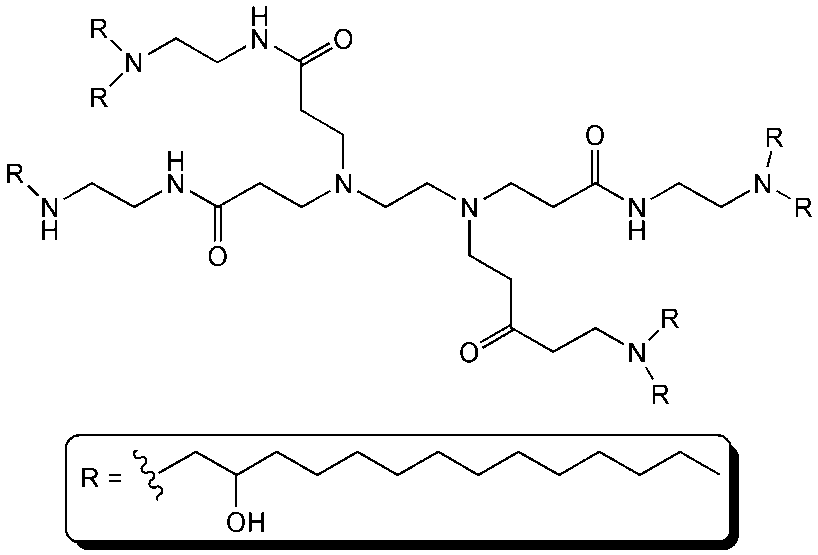


**Fig. S8.** Chemical structure of amphiphilic cationic lipid-like compound G0-C14.


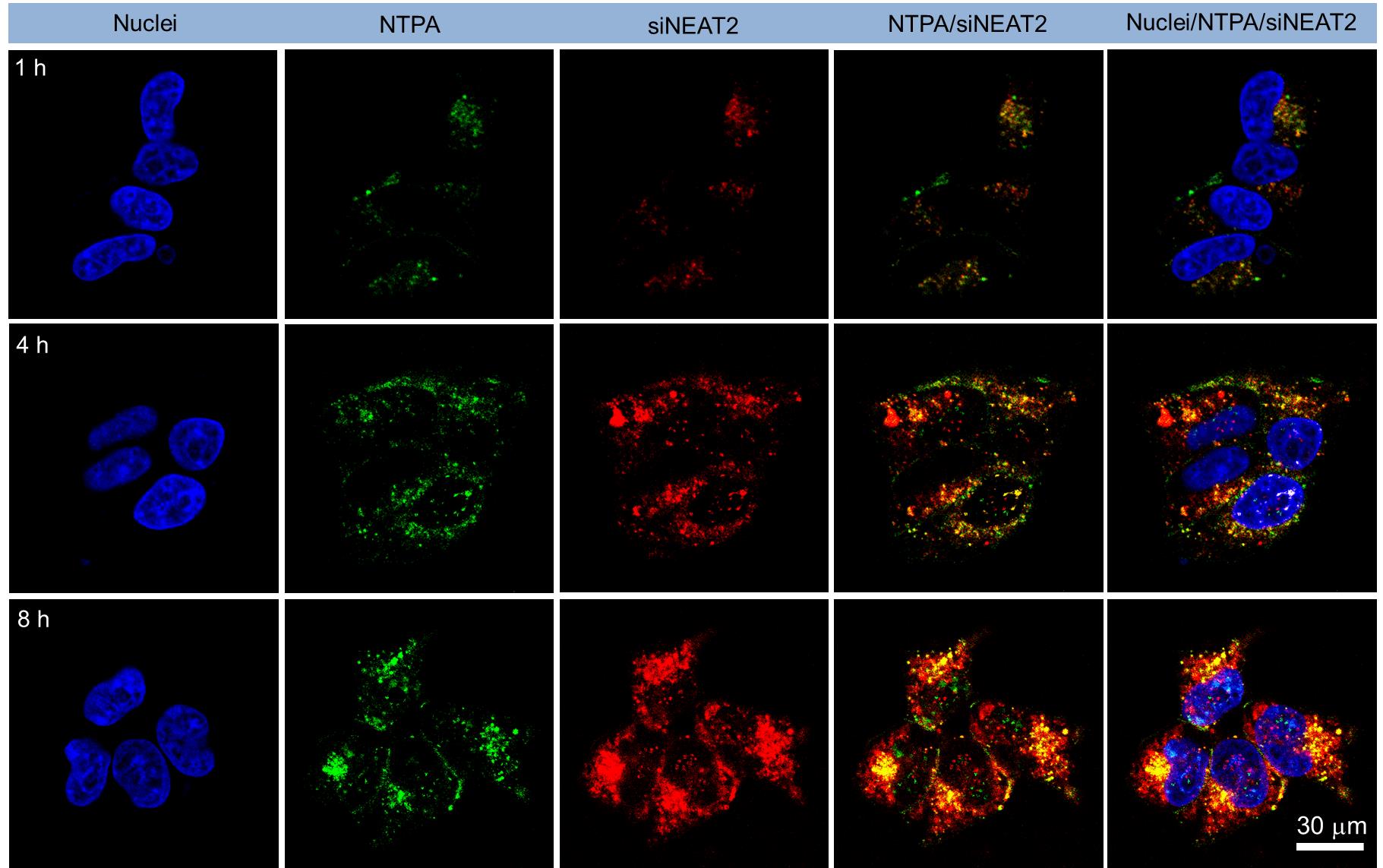


**Fig. S9.** CLSM images of SK-Hep1 cells incubated with the NPs20 for 1, 4, 8 hours at a siNEAT2 dose of 30 nM. Hoechst 33342 was used to stain the nuclei; NTPA and siNEAT2 were labeled with Alexa Fluor 488 and Cy5, respectively.

**
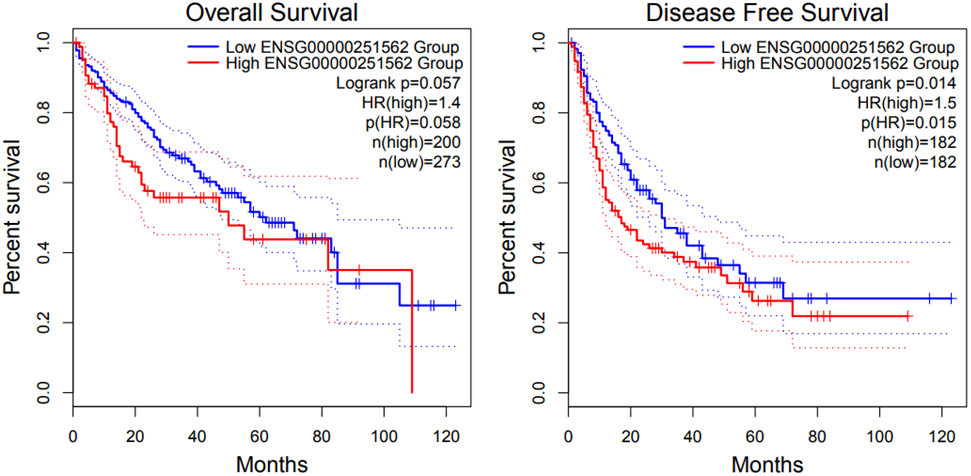
**

**Fig. S10.** TCGA database showing the overall survival (OS) and disease free survival (DFS) of liver cancer patients with high and low lncNEAT2 expression.

**
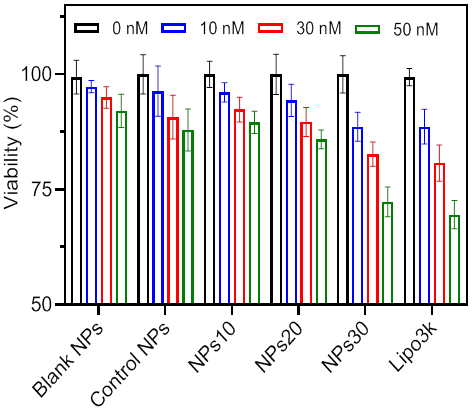
**

**Fig. S11.** Viability of SK-Hep1 cells incubated with the siRNA-free NPs (Blank NPs), Lipo3k/scrambled siRNA complexes, and the NPs10, NPs20, NPs30, and Control NPs loading scrambled siRNA for 48 h at different siRNA doses, respectively.





**Fig. S12.** Size and PDI of NPs20 incubated in PBS solution containing 10% FBS for different times.

**
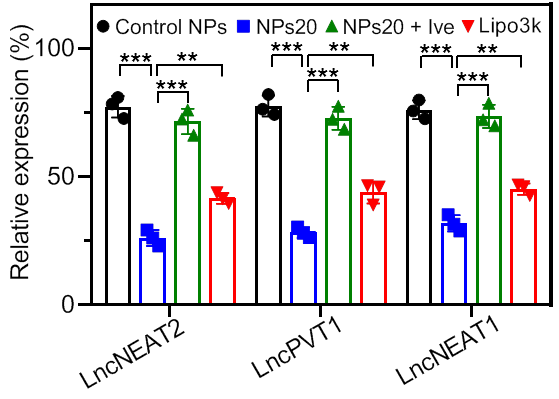
**

**Fig. S13.** Expression level of lncNEAT2, lncPVT1, and lncNEAT1 determined by qRT-PCR analysis of HepG2 cells treated with the NPs20, Control NPs, and Lipo3k/siRNA complexes at a siRNA dose of 30 nM.

**
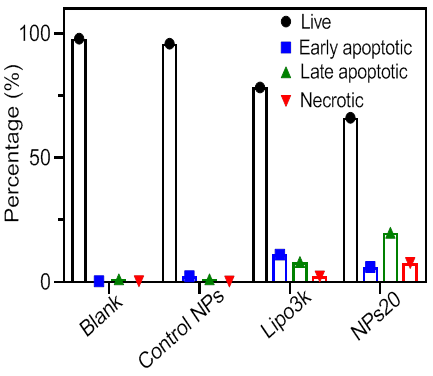
**

**Fig. S14.** Quantification of apoptosis determined by flow cytometry analysis of SK-Hep1 cells treated with the NPs20, Control NPs, and Lipo3k/siNEAT2 complexes at a siRNA dose of 30 nM.

**
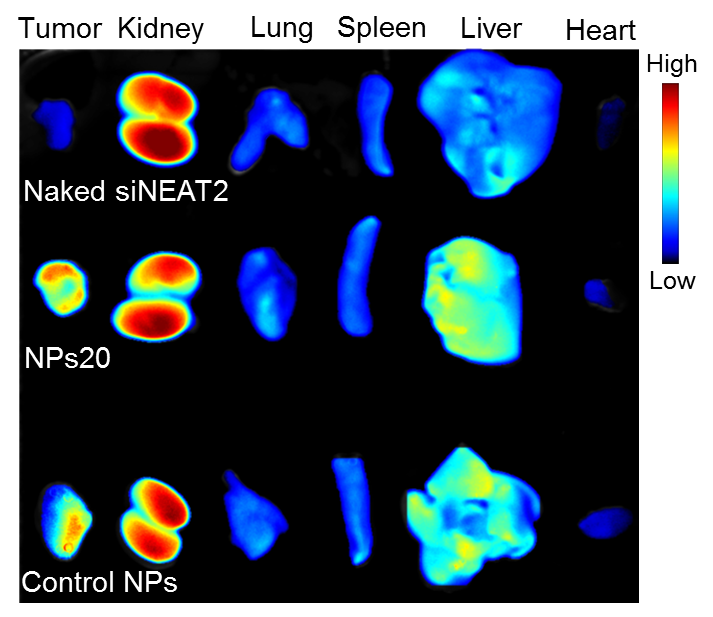
**

**Fig. S15.** Overlaid fluorescence image of tumors and main organs of SK-Hep1 xenograft tumor-bearing mice at 24 h post injection of naked siNEAT2, NPs20, and Control NPs.

**Fig. S16.** Body weight of SK-Hep1 xenograft tumor-bearing mice treated with PBS, naked siNEAT2, Control NPs, NPs20, and NPs20 loading scrambled siRNA (NC-NPs20).


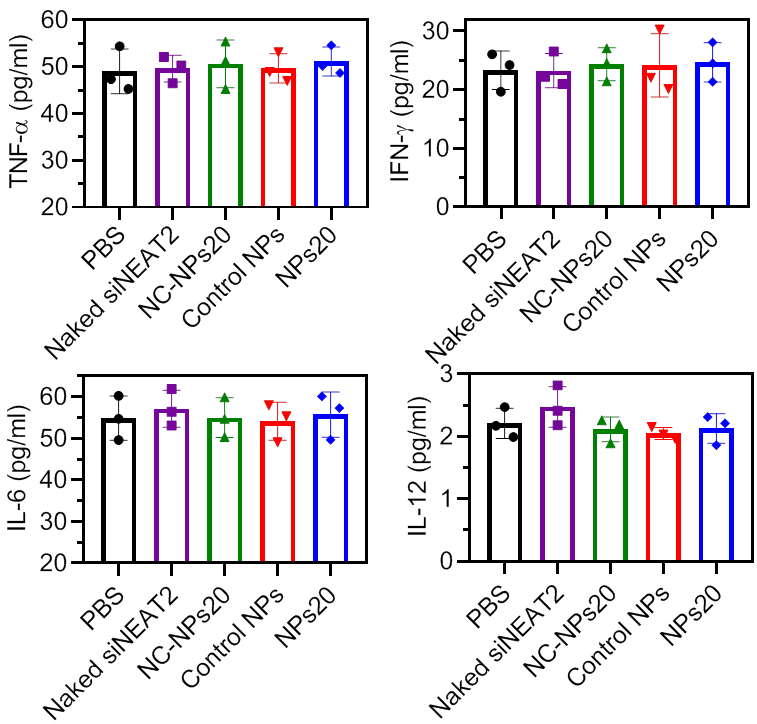


**Fig. S17.** Serum level of TNF-α, IFN-γ, IL-6, and IL-12 after three consecutive injections of PBS, naked siNEAT2, Control NPs, NPs20, and NC-NPs20.


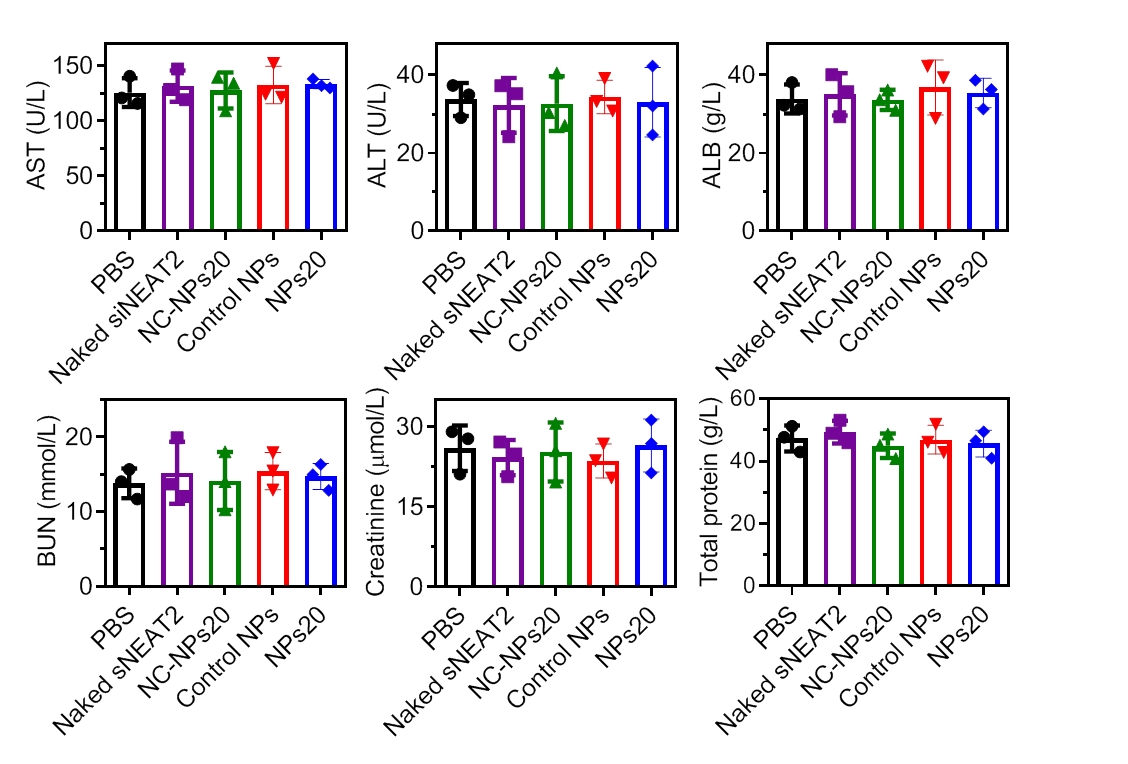


**Fig. S18.** Serum level of aspartate aminotransferase (AST), alanine aminotransferase (ALT), albumin (ALB), blood urine nitrogen (BUN), creatinine, and total protein after three consecutive injections of PBS, naked siNEAT2, Control NPs, NPs20, and NC-NPs20.

**
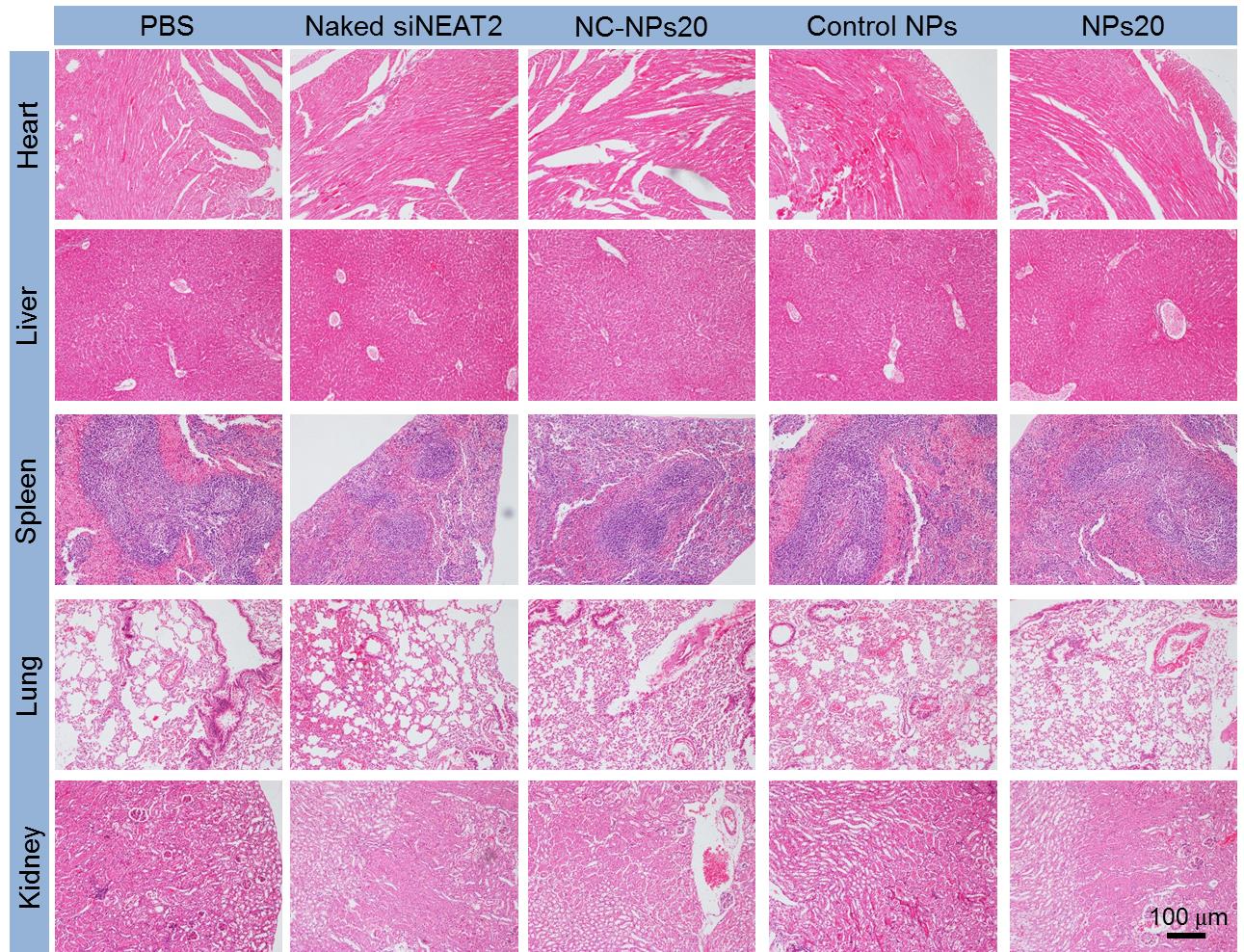
**

**Fig. S19.** Histological section of major organs of SK-Hep1 xenograft tumor-bearing mice treated with PBS, naked siNEAT2, Control NPs, NPs20, and NC-NPs20.
